# A double-duty champion: *Eurycoma longifolia* counters oxidative stress and unlocks autophagy to safeguard neurons and muscle cells

**DOI:** 10.1371/journal.pone.0355828

**Published:** 2026-08-10

**Authors:** Qing Huang, Shinong Gu, Hengzhi Deng, Annie George, Ashril Yusof

**Affiliations:** 1 Faculty of Sports and Exercise Science, Universiti Malaya, Kuala Lumpur, Malaysia; 2 College of Environment and Public Health, Xiamen Huaxia University, Xiamen, China; 3 Biotropics Malaysia Berhad, Selangor, Malaysia; Weill Cornell Medicine, UNITED STATES OF AMERICA

## Abstract

**Objectives:**

To investigate whether selected Malaysian plant extracts modulate the antioxidant-autophagy axis to enhance neuronal and skeletal muscle protection.

**Methods:**

Four standardized extracts (*Persicaria minor, Eurycoma longifolia* (EL)*, Labisia pumila,* and *Ipomoea aquatica*) were evaluated for antioxidant activity at 100, 200, and 300 μg/mL using ABTS, DPPH, and ORAC assays. The most active extract was further examined in SH-SY5Y neuronal-like cells and differentiated C2C12 myotubes. Cell viability was assessed using CCK-8, oxidative DNA damage was measured by 8-OHdG ELISA, and autophagy-related changes were evaluated by LC3/p62 immunofluorescence and western blot analysis of LC3-II and p62 with chloroquine (CQ) as a lysosomal inhibitor.

**Results:**

Among the four extracts, EL exhibited the strongest antioxidant activity, achieving the highest values in ABTS (107.59 ± 7.98 μmol Trolox/g), DPPH (27.28 ± 1.29 μmol Trolox/g), and ORAC (81.12 ± 4.81 μmol Trolox/g) at 300 μg/mL, also showing the lowest IC₅₀ (167.22, 97.64, and 67.90 μg/mL for ABTS, DPPH, and ORAC, respectively), with regression analyses confirming significant dose-response relationships (R² > 0.96, *p* < 0.05). CCK-8 analysis showed that EL at 100–300 μg/mL did not cause overt cytotoxicity in either cell model after 24 h treatment (*p* > 0.2), whereas the positive cytotoxicity control markedly reduced viability (*p* < 0.001). 8-OHdG ELISA showed that EL reduced oxidative DNA damage, with significant reductions at 200 and 300 μg/mL in both SH-SY5Y and C2C12 cells (*p* < 0.05). H_2_O_2_ markedly increased 8-OHdG levels in both models (*p* < 0.001). Immunofluorescence analysis showed concentration-dependent modulation of LC3 and p62 autophagy-related markers. In SH-SY5Y cells, treatment with EL induced a dose-dependent increase in LC3 puncta (*p* < 0.001) and a significant reduction in p62 fluorescence (*p* < 0.01). Similar trends were observed in C2C12 myotubes, where LC3 puncta formation was enhanced (*p* < 0.05), and p62 levels decreased (*p* < 0.01). Western blot validation further showed that EL increased LC3-II abundance and that EL + CQ further increased LC3-II accumulation compared with CQ alone, particularly at 300 μg/mL (*p* < 0.05). p62 decreased under basal EL treatment but showed a non-canonical decrease in the CQ-only group and was therefore interpreted cautiously.

**Conclusions:**

EL showed the strongest antioxidant activity among the tested Malaysian plant extracts and modulated autophagy-related cellular markers without overt cytotoxicity. These findings identify EL as a promising candidate for further investigation in oxidative stress- and proteostasis-related cellular protection.

## 1. Introduction

Plant-derived compounds are an important source of bioactive molecules that support cellular homeostasis, where their antioxidant activities are widely recognized as a central mechanism for cellular protection [1,2].Reactive oxygen species (ROS) disrupt redox balance and damage proteins, lipids, and nucleic acids [2,3]. Plant antioxidants can counteract oxidative stress by scavenging free radicals, limiting oxidative damage, and supporting molecular stability [4,5]. Increasingly, this role has been linked with autophagy, the lysosomal pathway responsible for recycling dysfunctional organelles and protein aggregates [6,7].

Redox imbalance exerts a profound influence on major regulatory nodes of the autophagy pathway [7], as exemplified by AMPK/mTOR/ULK1 [8], which integrates cellular energy sensing with autophagosome initiation, and by TFEB [9,10], which governs lysosomal biogenesis together with the transcription of autophagy-related genes. The Keap1/Nrf2/p62 axis further illustrates the interaction between antioxidant defences and selective autophagy [11,12],as p62 not only participates in substrate degradation but also regulates the stability of Nrf2 [13]. At the same time, functional autophagy feeds back to the redox system by removing damaged mitochondria through mechanisms such as PINK1/Parkin-mediated mitophagy and by supporting the degradation of oxidised proteins through the ubiquitin-proteasome system (UPS) and chaperone-mediated autophagy (CMA) pathways [10,14], thereby reducing intracellular sources of ROS [15]. Therefore, examining antioxidant activity and autophagy in parallel may provide a more comprehensive understanding of cellular protection.

Meanwhile, Malaysia’s exceptional biodiversity, together with a long and well-established tradition of ethnopharmacological practice, provides a rich foundation for exploring how plant-derived metabolites contribute to cellular protective mechanisms [1]. Within this ecological and cultural landscape, many native edible and medicinal species are not only widely consumed as part of daily diets or traditional remedies but are also regarded as safe and culturally accepted [16]. These plants are notable for their high content of bioactive metabolites, including flavonoids [17], phenolics [18], and quassinoids with known antioxidant and anti-inflammatory effects [19].

Although these species show promising antioxidant activity, it remains unknown whether they modulate autophagy, a question whose answer could reveal further mechanisms behind their health benefits. We therefore selected four representative species for a comparative study. Among them, *Eurycoma longifolia* (Tongkat Ali) is particularly noteworthy for its distinctive phytochemistry. As an emblematic medicinal plant of the region used as a restorative tonic [20], and its root is rich in highly oxygenated quassinoids (e.g., eurycomanone, eurycomalactone) and β-carboline alkaloids [20,21]. This unique compositional profile suggests a strong inherent capacity for redox modulation. *Persicaria minor* (kesum) is a fragrant culinary herb that contributes both flavor and health benefits to Malaysian cuisine [22], and is rich in flavonoids and rosmarinic acid associated with antioxidant and cognitive benefits [23,24]. *Labisia pumila* (Kacip Fatimah) is prescribed in women’s health and provides phenolics with antioxidant and anti-inflammatory properties [25]. *Ipomoea aquatica* (kangkung or water spinach), a dietary staple, contributes polyphenols with well-documented antioxidant activity and the advantage of widespread safety [26,27]. Building on this background, the present study undertook a comparative evaluation of four standardized Malaysian plant extracts under identical experimental conditions to reduce selection bias and to allow a more objective assessment of their biological potential.

Chemical antioxidant assays are useful for ranking radical-scavenging activity, but they do not directly establish intracellular antioxidant effects. Furthermore, to determine whether chemical antioxidant activity translates into biological outcomes, appropriate cellular readouts are required. LC3 and p62 were chosen because they capture complementary aspects of the autophagy process [8]. LC3 puncta reflect the formation of autophagosomes, whereas p62 serves as an adaptor and substrate whose reduction indicates cargo degradation [8,28]. At the same time, LC3 or p62 changes alone are insufficient to establish complete autophagic flux because increased LC3 signal may reflect either enhanced autophagosome formation or impaired degradation. For this reason, the study includes Western blot analysis of LC3-II and p62 in the presence of chloroquine (CQ), a lysosomal inhibitor, to evaluate LC3-II turnover under lysosomal inhibition [29,30].

To address these questions, SH-SY5Y neuronal-like cells and differentiated C2C12 myotubes were selected because neuronal and muscle cell models are highly relevant to redox imbalance, proteostatic stress, and age-related functional decline [31,32]. Antioxidant activity of standardized Malaysian plant extracts was quantified using ABTS, DPPH, and ORAC assays. Based on the screening results, one extract was selected for further cell-based validation in SH-SY5Y and C2C12 cells. Autophagy modulation was assessed by immunofluorescence analysis of LC3 puncta and p62 degradation, and CQ-based Western blot analysis of LC3-II and p62. By linking chemical assays with cellular outcomes in neuronal and muscular contexts, the study sought to determine whether these extracts can reinforce redox balance, activate autophagy, and contribute to cellular resilience.

## 2. Materials and methods

### 2.1 Plant extracts and materials

All standardized Malaysian plant extracts, including *Persicaria minor* (PM, Batch No. KE240902), *Eurycoma longifolia* (EL, Batch No. TA2401002), *Labisia pumila* (LP, Batch No.KE241005P), and *Ipomoea aquatica* (IA, Batch No. P24/RE025), were obtained from Biotropics Malaysia Berhad, Malaysia. Certificates of Analysis for the standardized plant extracts are publicly available in the Figshare repository (https://doi.org/10.6084/m9.figshare.33006623). The anonymized Outgoing Material Transfer Agreement is provided in Supporting Appendix.

Antioxidant activity assays and in vitro cell experiments were both conducted at Xiamen Huaxia University, Xiamen, China. The detailed materials and procedures used in this study are available at protocols.io (https://dx.doi.org/10.17504/protocols.io.kxygxrk7og8j/v1)

### 2.2 Establishment of neuronal-like SH-SY5Y and skeletal muscle C2C12 models

SH-SY5Y human neuroblastoma cells and C2C12 mouse myoblast cells were selected as representative in vitro models for this study [31,32]. These cell types are particularly relevant in the context of ageing, as both neurons and skeletal muscle are post-mitotic tissues highly vulnerable to the accumulation of oxidative damage and dysregulated autophagy, key drivers of functional decline [33,34].

#### 2.2.1 Culture and treatment of SH-SY5Y cells.

The human neuroblastoma cell line SH-SY5Y (ATCC^®^ CRL-2266™) was cultured in DMEM/F-12 (Gibco, USA) supplemented with 10% FBS (Gibco, USA) and 1% penicillin-streptomycin and maintained at 37 °C in a humidified incubator with 5% CO_2_. For neuronal differentiation, cells were seeded at a density of 1 × 10^4^ cells/mL and allowed to attach overnight. The medium was then replaced with differentiation medium consisting of DMEM/F-12 supplemented with 2% FBS and 10 μM all-trans RA (Sigma-Aldrich, USA), and cells were cultured for 4 days with medium replacement every 48 h. Differentiation was confirmed by neurite outgrowth observed under phase-contrast microscopy. Following differentiation, cells were treated with standardized Eurycoma longifolia (EL) extract. Four groups were established: (i) 0 μg/mL (control), (ii) 100 μg/mL, (iii) 200 μg/mL, and (iv) 300 μg/mL. EL extract was added to the culture medium at the indicated final concentrations and incubated for 24 h before subsequent analyses.

#### 2.2.2 Differentiation and treatment of C2C12 cells.

Murine skeletal muscle myoblasts C2C12 (ATCC^®^ CRL-1772™) were cultured in DMEM (4.5 g/L D-glucose) supplemented with 10% FBS and 1% penicillin-streptomycin and maintained at 37 °C in a humidified incubator with 5% CO_2_. Cells were seeded at a density of approximately 1 × 10^4^ cells/mL in 24-well plates, upon reaching 90–100% confluence, the growth medium was replaced with differentiation medium (DMEM supplemented with 2% horse serum). The medium was refreshed every 48 h for 4–6 days until the formation of multinucleated myotubes and the establishment of a stable myotube network was confirmed under phase-contrast microscopy, thereby generating a skeletal muscle cell model. Based on antioxidant screening results, standardized EL extract was selected for treatment. Cells were divided into four groups: (i) 0 μg/mL (control), (ii) 100 μg/mL, (iii) 200 μg/mL, and (iv) 300 μg/mL. EL was added to the differentiation medium at the designated concentrations and incubated for 24 h.

### 2.3 CCK-8 cell viability assay

Cell viability was assessed using the CCK-8 assay to determine whether EL treatment caused overt cytotoxicity under the experimental conditions used for subsequent assays. SH-SY5Y and C2C12 cells in the logarithmic growth phase were seeded into 96-well plates at 3,000 cells per well in 100 μL culture medium and allowed to recover overnight at 37 °C in a humidified incubator with 5% CO_2_. Cells were then treated with EL at 100, 200, and 300 μg/mL for 24 h. A positive cytotoxicity control was included. After treatment, 10 μL of CCK-8 reagent (SuperKine™ Enhanced Cell Counting Kit-8, BMU106-CN, Abbkine, China) was added to each well, and plates were incubated at 37 °C for 45 min. Absorbance was measured at 450 nm using a microplate reader (DNM-9602, Beijing Perlong New Technology, China). Blank wells containing medium and CCK-8 reagent without cells were used for background correction. Relative cell viability was calculated as a percentage of the negative control group. Three biological replicates were analyzed, with technical replicates included within each experiment.

### 2.4 Measurement of ABTS radical scavenging activity

ABTS• ⁺ radical cation was generated by mixing 7 mmol/L ABTS with 2.45 mmol/L potassium persulfate (K_2_S_2_O₈) at a 1:1 ratio and incubating the mixture in the dark at room temperature for 12–16 h. The resulting stock solution was diluted with 10 mmol/L phosphate buffer (PB) (pH 7.4) to an absorbance of 0.70 ± 0.02 at 734 nm, yielding the working solution. For the assay, 100 μL of ABTS• ⁺ working solution was mixed with 100 μL of each plant extract solution (final concentrations: 100, 200, and 300 μg/mL) in a 96-well plate. Each concentration was tested in triplicate (n = 3), starting from the lowest concentration. After gentle mixing and incubation in the dark at room temperature for 10 min, absorbance was measured at 734 nm using a microplate spectrophotometer. Controls included ABTS working solution with solvent (A₀) and extract solution with solvent (Ar). The scavenging activity (%) was calculated as:


$$\begin{array}{c} \text{Scavenging activity }(\%)=[\text{1}-(\text{At}-\text{Ar})/{\text{A}}_{\text{0}}]\times \text{100} \end{array}$$

where At is the absorbance of the reaction mixture. The half-maximal scavenging concentration (IC₅₀) was obtained from the concentration-response regression. Trolox was used as the reference standard, and results were expressed as Trolox equivalent antioxidant capacity (TEAC, μmol Trolox equivalents per g extract) (mean ± SD).

### 2.5 Measurement of DPPH radical scavenging activity

DPPH solution was prepared by dissolving 6.19 mg DPPH in methanol and adjusting the volume to 50 mL (2.0 × 10^−4^ mol/L), protected from light until use. For the assay, 100 μL of each plant extract solution (final concentrations: 100, 200, and 300 μg/mL) was mixed with 100 μL of the DPPH solution in a 96-well plate. Each concentration was tested in triplicate (n = 3), starting from the lowest concentration. After gentle mixing, the plate was incubated in the dark at room temperature for 30 min, and absorbance was measured at 517 nm using a microplate spectrophotometer. Controls included DPPH solution with solvent (A₀) and extract solution with solvent (Ar). Radical scavenging activity (%) was calculated as:


$$\begin{array}{c} \text{Scavenging activity }(\%)=[\text{1}-(\text{At}-\text{Ar})/{\text{A}}_{\text{0}}]\times \text{100} \end{array}$$

where At is the absorbance of the reaction mixture. The half-maximal scavenging concentration (IC₅₀) was obtained from the concentration-response regression. Trolox was used as the reference antioxidant, and results were expressed as Trolox equivalent antioxidant capacity (TEAC, μmol Trolox equivalents per g extract) (mean ± SD).

### 2.6 Assessment of ORAC antioxidant capacity

ORAC assay was performed in black 96-well microplates. Briefly, 20 μL of PBS (control), Trolox standard solution, or plant extract solution (final concentrations: 100, 200, and 300 μg/mL) was added to each well in triplicate (n = 3). Then, 200 μL of fluorescein solution (0.96 μmol/L, prepared in PBS) was added, and the plate was incubated at 37 °C for 20 min with intermittent shaking. Subsequently, 20 μL of freshly prepared 119 mmol/L AAPH [2,2’-azobis(2-methylpropionamidine) dihydrochloride] solution was rapidly added to initiate the reaction. Fluorescence was monitored using a microplate reader at 37 °C with an excitation wavelength of 485 nm and an emission wavelength of 528 nm. Readings were taken every 4.5 min for 2.5 h, with 5 s shaking before each measurement. The area under the fluorescence decay curve (AUC) was calculated for each sample and compared with the Trolox calibration curve. ORAC values were expressed as μmol Trolox equivalents per g extract (mean ± SD).

### 2.7 Measurement of 8-OHdG levels

Oxidative DNA damage was assessed by measuring 8-hydroxy-2′-deoxyguanosine (8-OHdG) levels in SH-SY5Y and C2C12 cells after EL treatment. Cells were assigned to five groups: control, EL 100 μg/mL, EL 200 μg/mL, EL 300 μg/mL, and H_2_O_2_ was applied at 100 μM for 24 h as the positive oxidative-damage control. After treatment, cell lysates were collected, and 8-OHdG levels were quantified using a commercial ELISA kit (Elabscience E-EL-0028, China) according to the manufacturer’s instructions. Absorbance was measured using a microplate reader (DNM-9602, Beijing Perlong New Technology, China), and 8-OHdG concentrations were calculated from the standard curve. Data are presented as mean ± SD from three biological replicates. H_2_O_2_ was included as a positive oxidative-damage control to verify assay responsiveness.

### 2.8 Immunofluorescence staining for autophagy markers

After treatment, SH-SY5Y and C2C12 cells were fixed with 4% paraformaldehyde at room temperature for 15 min and rinsed three times with PBS. Cells were permeabilized with 0.1% Triton X-100 in PBS for 10 min, followed by blocking with 3% BSA in PBS for 1 h at room temperature. Primary antibodies against LC3B (rabbit monoclonal, 1:200; Cell Signaling Technology, USA, #2775) and p62 (mouse monoclonal, 1:200; Cell Signaling Technology, USA, 39749S) were applied overnight at 4 °C in a humidified chamber. The next day, cells were washed with PBS and incubated with species-appropriate Alexa Fluor-conjugated secondary antibodies (1:500, Thermo Fisher Scientific) for 1 h at 37 °C in the dark. Nuclei were counterstained with 1 μg/mL DAPI for 10 min, followed by three washes with PBS. Fluorescent images were acquired using a confocal laser scanning microscope (Leica Microsystems, Germany), and quantitative analysis of LC3 puncta formation and p62 fluorescence intensity was performed using ImageJ (NIH, USA) software with consistent threshold settings across all groups under blinded group coding, specifically, the LC3 puncta area was thresholder and quantified as the number of LC3 puncta per cell for at least 50 cells per condition.

### 2.9 Western blot analysis of LC3-II and p62 under lysosomal inhibition

SH‑SY5Y and C2C12 cells were assigned to six groups: control, EL 100 μg/mL, EL 300 μg/mL, CQ 50 μM, EL 100 + CQ, and EL 300 + CQ. After treatment, cells were lysed in RIPA buffer (Cat# R002, Solarbio, China) supplemented with PMSF (Cat# P0100, BOSTER, China). Protein concentration was determined using a Bradford assay kit (AR0145, BOSTER, China) with BSA standards. Equal amounts of protein (30 μg) were mixed with 4 × loading buffer, boiled for 5 min, separated by 10% SDS‑PAGE (gel preparation kit AR0138, BOSTER, China), and transferred to PVDF membranes (Cat# IPVH00010, Millipore, USA) in Tris‑glycine transfer buffer (AR0141, BOSTER, China) at 25 V for 30–40 min. Membranes were blocked with 5% non‑fat milk in TBST for 1 h at room temperature, then incubated overnight at 4 °C with primary antibodies against LC3‑II (#AF4650, 1:1000, Affinity, China), p62 (#AF5384, 1:1000, Affinity, China), and β‑actin (#AF7018, 1:5000, Affinity, China). After washing with TBST, membranes were incubated with HRP‑conjugated secondary antibody (ab7090, 1:8000, Abcam, UK) for 1 h. Protein bands were visualized using Western Lightning™ Chemiluminescence Reagent (NEL10300EA, PerkinElmer, USA) and imaged with a FluorChem FC2 system. Band intensities were quantified by densitometry. LC3‑II and p62 were normalized to β‑actin, and ΔLC3‑II was calculated as the increase in relative LC3‑II in the EL + CQ groups compared with the CQ‑only group, indicating autophagic flux.

### 2.10 Statistical analysis

All quantitative data are presented as mean ± SD. Statistical analyses were performed using GraphPad Prism 9.0 and SPSS 26.0. For antioxidant assays, concentration-response relationships were assessed by regression analysis, and IC₅₀ values were calculated using a four-parameter logistic model where applicable. For assays in which multiple treatment groups were compared with one shared control group, one-way ANOVA followed by Dunnett’s post hoc test was used. Western blot data were normalized to β-actin and analyzed by one-way ANOVA with Šídák-corrected planned comparisons. EL and CQ groups were compared with control, while EL + CQ groups were compared with CQ.Image quantification was performed using consistent threshold settings under blinded group coding.

For all analyses, *p* < 0.05 was considered statistically significant. Significance levels were reported as * *p* < 0.05, ** *p* < 0.01, and *** *p* < 0.001. Non-significant comparisons were reported as n.s.

## 3. Results

### 3.1 EL did not induce overt cytotoxicity in SH-SY5Y and C2C12 cells

**Fig 1:**
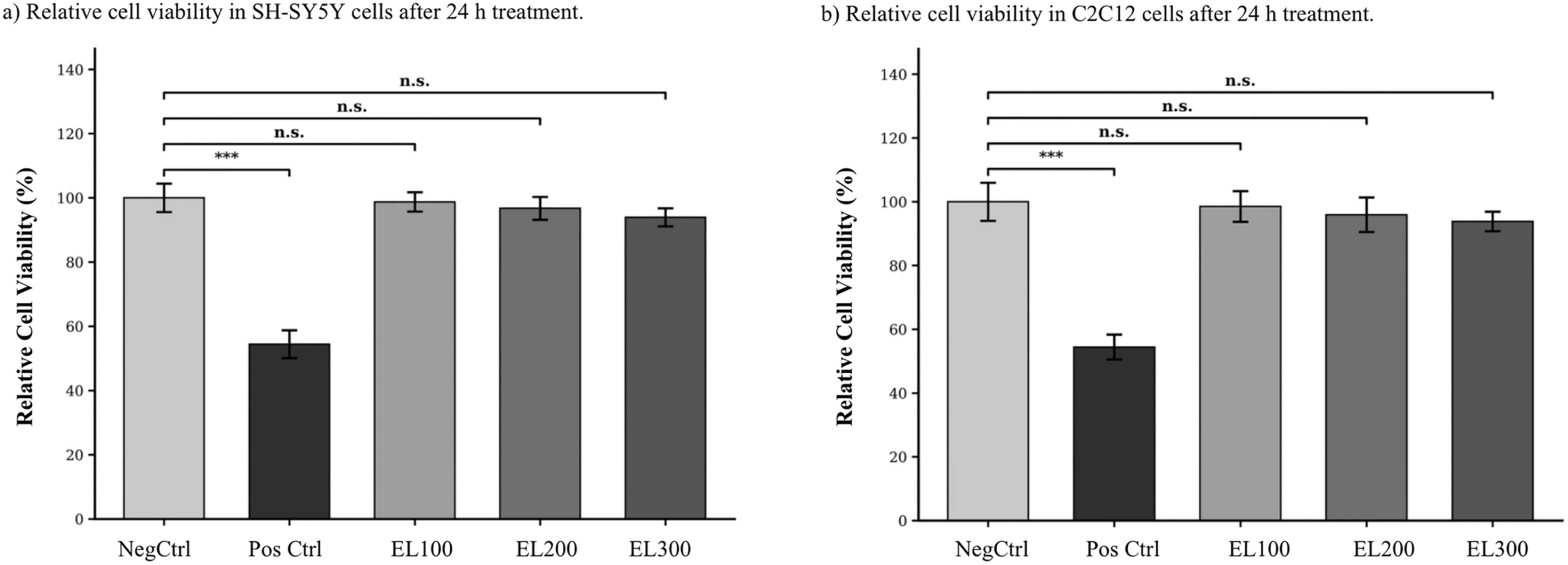
Relative cell viability in SH-SY5Y and C2C12 cells. (a) Relative cell viability in SH-SY5Y cells after 24 h treatment. (b) Relative cell viability in C2C12 cells after 24 h treatment. Cell viability was determined using the CCK-8 assay and expressed as a percentage of the negative control group. Data are presented as mean ± SD from three biological replicates. Statistical analysis was performed using one-way ANOVA followed by Dunnett’s post hoc test versus the negative control group. * *p* < 0.05, **p < 0.01, *** *p* < 0.001; n.s., not significant.

Cell viability was evaluated using the CCK-8 assay to determine whether EL treatment caused overt cytotoxicity under the experimental conditions used for subsequent assays (Fig 1 and S1 Table). In SH-SY5Y cells, the positive cytotoxicity control reduced relative cell viability to 54.43 ± 4.32% of the negative control (*p* < 0.001). In contrast, EL treatment-maintained viability at 98.73 ± 3.00%, 96.74 ± 3.56%, and 93.94 ± 2.83% at 100, 200, and 300 μg/mL, respectively, with no significant reduction compared with the negative control. A similar pattern was observed in C2C12 cells, where the positive cytotoxicity control reduced viability to 54.43 ± 3.92% (*p* < 0.001), while EL treatment-maintained viability at 98.54 ± 4.80%, 95.93 ± 5.43%, and 93.81 ± 3.06% at 100, 200, and 300 μg/mL, respectively. These findings indicate that the tested EL concentrations did not cause overt cytotoxicity after 24 h exposure.

### 3.2 Comparative antioxidant activity of plant extracts by ABTS, DPPH, and ORAC assays

**Fig 2:**
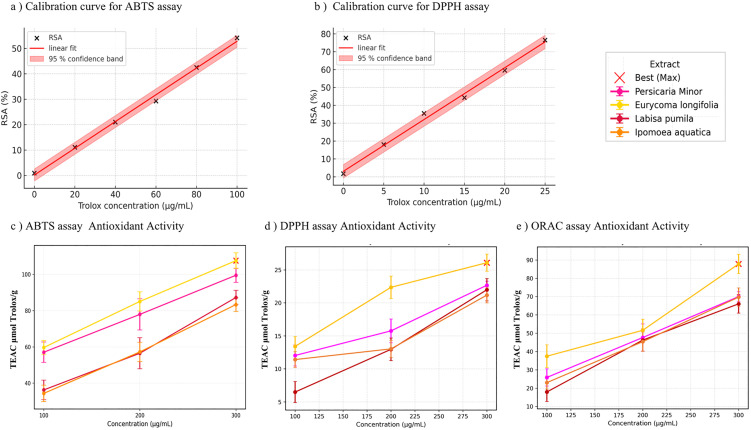
Standard curves and antioxidant activity comparisons of plant extracts using three assays. (a) Calibration curve for ABTS assay using Trolox (0-100 μg/mL) as the standard, showing a linear fit (red line), mean ± SD (black crosses), and 95% confidence band (shaded area). (b) Calibration curve for DPPH assay using Trolox (0-25 μg/mL), under the same conditions. To minimize variability due to environmental factors (e.g., light, temperature), calibration was performed under the same day-specific spectrophotometric conditions. (c-e) TEAC values (Trolox Equivalent Antioxidant Capacity) of four plant extracts (*Persicaria Minor*, *Eurycoma longifolia*, *Labisia pumila*, and *Ipomoea aquatica*) at three concentrations (100, 200, 300 μg/mL) assessed by ABTS (c), DPPH (d), and ORAC (e) assays. Error bars represent standard deviation (SD), and red “X” denotes the highest antioxidant activity (best-performing extract per assay) selected for subsequent validation.

Prior to evaluating the antioxidant activity of the plant extracts, calibration curves were established using Trolox as the reference standard. Both the ABTS (0–100 µg/mL) and DPPH (0–25 µg/mL) assays exhibited strong linearity between Trolox concentration and radical scavenging activity (R² > 0.99), with narrow 95% confidence intervals, indicating reliable analytical performance (Fig 2a and 2b). These standard curves were used to calculate TEAC values of the plant extracts, thereby ensuring comparability across assays.

#### 3.2.1 ABTS radical scavenging activity.

The ABTS results are summarized in S2 Table and Fig 2c. All four extracts demonstrated significant concentration-dependent increases in scavenging activity at 100, 200, and 300 µg/mL (R² > 0.97). EL exhibited the strongest activity, reaching 107.59 ± 7.98 µmol Trolox/g at 300 µg/mL, with the lowest IC₅₀ value (167.22 µg/mL, *p* = 0.020). In contrast, PM, LP, and IA displayed weaker effects, with IC₅₀ values exceeding 450 µg/mL. These results suggest that EL possesses superior electron-donating capacity, possibly due to its enrichment in bioactive polyphenolics.

#### 3.2.2 DPPH radical scavenging activity.

The DPPH assay results are summarized in S3 Table and Fig 2d. EL again showed the highest activity among the four extracts, reaching 27.28 ± 1.29 µmol Trolox/g at 300 µg/mL, with the lowest IC₅₀ (97.64 µg/mL). However, the regression slope did not reach statistical significance (*p* = 0.113), possibly due to the narrower concentration range of this assay. PM and LP exhibited moderate activity (IC₅₀ = 297.41 µg/mL and 312.14 µg/mL, respectively), while IA remained the weakest (IC₅₀ = 313.06 µg/mL). Therefore, although the DPPH dose-response trend should be interpreted cautiously, EL still showed the strongest hydrogen-donating potential under the tested conditions.

#### 3.2.3 ORAC antioxidant activity.

The ORAC assay (S4 Table and Fig 2e) further validated the superior antioxidant capacity of EL. At 300 µg/mL, EL achieved 81.12 ± 4.81 µmol Trolox/g, corresponding to the lowest IC₅₀ (67.90 µg/mL, *p* = 0.002), which was significantly more potent than the other extracts. LP also showed notable activity (IC₅₀ = 83.72 µg/mL, *p* = 0.023), whereas PM and IA exhibited much higher IC₅₀ values (339.42 µg/mL and 480.96 µg/mL, respectively). Given that the ORAC assay measures peroxyl radical neutralization through hydrogen atom transfer, these findings highlight the robust radical-quenching capacity of EL.

In combination, the results from ABTS, DPPH, and ORAC assays consistently demonstrated a strong dose-dependent scavenging effect across all extracts (R² > 0.95). Among the tested species, EL exhibited the lowest IC₅₀ values and the highest activity in all three assays, supporting its stronger antioxidant potential, and may be attributed to the standardized bioactive components in the extract, notably the quassinoid eurycomanone (1.09%) and high glycosaponin content (57.1%), both of which have been independently associated with antioxidant and cell-modulating properties. LP showed intermediate activity, while PM and IA were markedly less effective.

### 3.3 EL reduced 8-OHdG levels in SH-SY5Y and C2C12 cells

To further evaluate intracellular oxidative damage, 8-OHdG levels were measured in SH-SY5Y and C2C12 cells after EL treatment (S5 Table). In SH-SY5Y cells, 8-OHdG levels were 2.98 ± 0.15 ng/mL in the control group. EL100 showed a decreasing trend but did not reach statistical significance (2.60 ± 0.11 ng/mL, *p* = 0.055), whereas EL200 and EL300 significantly reduced 8-OHdG levels to 2.45 ± 0.12 ng/mL (*p* = 0.009) and 2.33 ± 0.17 ng/mL (*p* = 0.002), respectively. H_2_O_2_ markedly increased 8-OHdG levels to 6.11 ± 0.24 ng/mL (*p* < 0.001). In C2C12 cells, 8-OHdG levels were 2.91 ± 0.14 ng/mL in the control group. EL100 showed a non-significant decreasing trend (2.57 ± 0.13 ng/mL, *p* = 0.062), whereas EL200 and EL300 significantly reduced 8-OHdG levels to 2.45 ± 0.11 ng/mL (*p* = 0.014) and 2.33 ± 0.15 ng/mL (*p* = 0.003), respectively. H_2_O_2_ increased 8-OHdG levels to 5.86 ± 0.21 ng/mL (*p* < 0.001). These results support that EL attenuated oxidative DNA damage in both cell models.

### 3.4 Immunofluorescence analysis of autophagy markers in SH‑SY5Y and C2C12 cells

**Fig 3:**
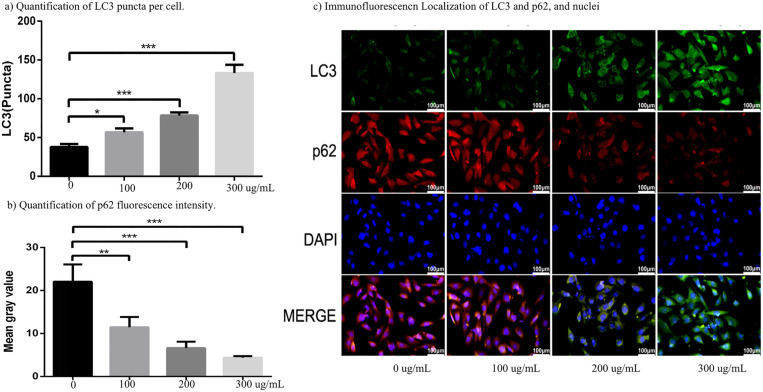
Immunofluorescence analysis of LC3 and p62 in SH-SY5Y cells. (a) Quantification of LC3 puncta per cell. (b) Quantification of p62 fluorescence intensity. (c) Immunofluorescence localization of LC3 and p62, and nuclei, showing LC3, p62, and DAPI staining after 24 h treatment with EL at 0, 100, 200, and 300 μg/mL. EL treatment increased LC3 puncta formation and reduced p62 fluorescence intensity in a concentration-dependent manner, indicating modulation of autophagy-related markers. Scale bar = 100 μm. *p < 0.05, ** *p* < 0.01, *** *p* < 0.001 vs. 0 μg/mL group.

**Fig 4:**
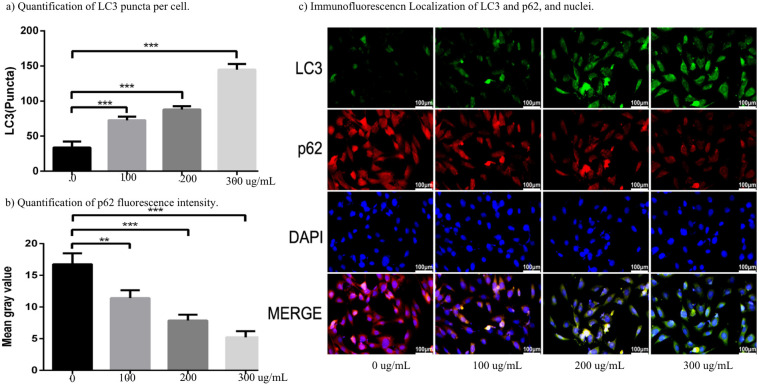
Immunofluorescence analysis of LC3 and p62 in C2C12 cells. (a) Quantification of LC3 puncta per cell. (b) Quantification of p62 fluorescence intensity. (c) Immunofluorescence localization of LC3 and p62, and nuclei, showing LC3, p62, and DAPI staining after 24 h treatment with EL at 0, 100, 200, and 300 μg/mL. EL treatment increased LC3 puncta formation and reduced p62 fluorescence intensity in a concentration-dependent manner, supporting autophagy-related marker modulation in C2C12 myotubes. Scale bar = 100 μm. * *p* < 0.05, ** *p* < 0.01, *** *p* < 0.001 vs. 0 μg/mL group.

Immunofluorescence analysis revealed marked changes in autophagy markers after exposure to EL extract in both cell lines. In SH‑SY5Y cells (Fig 3), quantification of LC3 puncta showed a progressive, dose‑dependent increase across treatment groups. At 100 μg/mL, the number of puncta was already significantly higher than the control (*p* < 0.001), with further increases at 200 and 300 μg/mL (*p* < 0.001). In parallel, p62 fluorescence intensity decreased gradually, with significant reductions at 100 μg/mL (*p* < 0.01) and further declines at 200 and 300 μg/mL (*p* < 0.001). Morphologically, LC3 staining shifted from faint and diffuse in control cells to bright, punctate signals in treated groups, while p62 staining became progressively weaker. In merged images, the overlap of LC3 and p62 signals decreased at higher concentrations, indicating enhanced autophagic activity. A similar pattern was observed in C2C12 myotubes (Fig 4). LC3 puncta per cell increased dose‑dependently, with significant differences at 100 μg/mL (*p* < 0.05) and highly significant at 200 and 300 μg/mL (*p* < 0.001). Conversely, p62 fluorescence intensity decreased stepwise, with significant reductions at 100 μg/mL (*p* < 0.01) and more pronounced decreases at 200 and 300 μg/mL (*p* < 0.001). Untreated myotubes exhibited diffuse LC3 distribution and strong p62 staining, whereas treated cells showed enhanced punctate LC3 signals and diminished p62 staining. The reduction in yellow overlap regions at higher concentrations further supported autophagic flux activation.

### 3.5 Western blot validation of LC3-II and p62 under lysosomal inhibition

**Fig 5:**
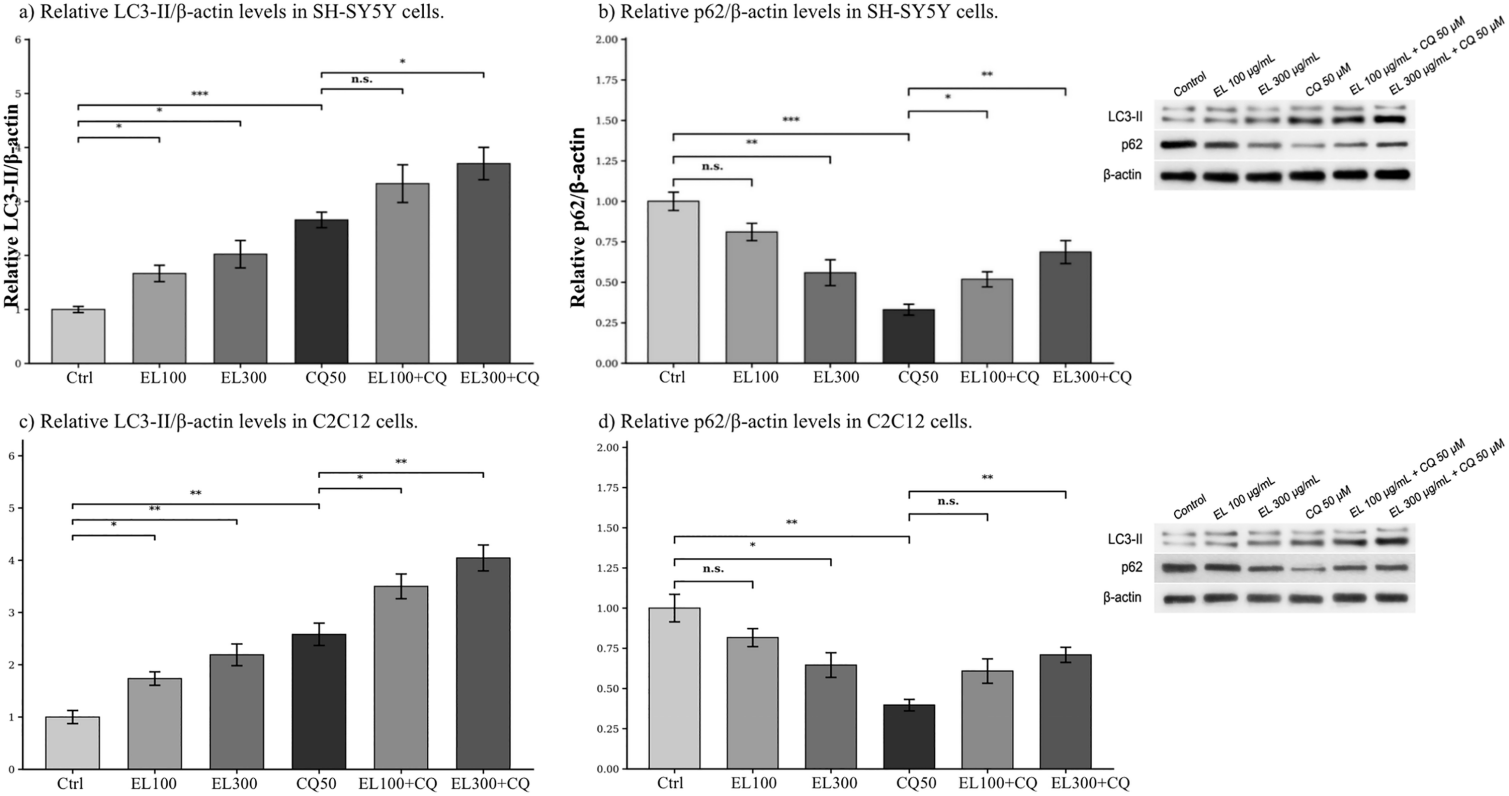
Western blot analysis of autophagy-related proteins in SH-SY5Y and C2C12 cells. (a) Relative LC3-II/β-actin levels in SH-SY5Y cells. (b) Relative p62/β-actin levels in SH-SY5Y cells. (c) Relative LC3-II/β-actin levels in C2C12 cells. (d) Relative p62/β-actin levels in C2C12 cells. LC3-II and p62 band intensities were normalized to β-actin and expressed relative to the control group. Data are presented as mean ± SD from three biological replicates. Statistical analysis was performed using one-way ANOVA followed by Šídák-corrected planned comparisons. Under basal conditions, EL-treated groups and the CQ-only group were compared with the control group. Under lysosomal inhibition, EL + CQ groups were compared with the CQ-only group. * *p* < 0.05, ** *p* < 0.01, ***P < 0.001; n.s., not significant.

In SH-SY5Y cells (Fig 5a–5c and S6 Table), EL treatment increased relative LC3-II/β-actin levels compared with the control group, with significant increases at both 100 and 300 μg/mL (*p* < 0.05). CQ alone markedly increased LC3-II accumulation (*p* < 0.001), confirming effective lysosomal inhibition. Under CQ treatment, EL100 + CQ showed a further but non-significant increase in LC3-II compared with CQ alone, whereas EL300 + CQ significantly increased LC3-II accumulation (*p* < 0.05). EL300 also significantly reduced p62 levels compared with the control group (*p* < 0.01). However, because p62 decreased in the CQ-only group, p62 was interpreted cautiously as a supportive autophagy-related marker rather than definitive evidence of autophagic flux.

A similar pattern was observed in C2C12 cells (Fig 5d–5f and S6 Table). EL significantly increased LC3-II levels under basal conditions, with stronger effects at 300 μg/mL (*p* < 0.01). CQ alone also significantly elevated LC3-II compared with the control group (*p* < 0.01). Importantly, both EL100 + CQ and EL300 + CQ further increased LC3-II accumulation compared with CQ alone (*p* < 0.05 and *p* < 0.01, respectively), supporting enhanced LC3-II turnover under lysosomal inhibition. For p62, EL300 significantly reduced basal p62 levels (*p* < 0.05), while EL300 + CQ significantly increased p62 compared with CQ alone (*p* < 0.01). Overall, these results support EL-mediated modulation of LC3-II turnover and p62-associated autophagy marker profiles, with the most consistent CQ-dependent effect observed at 300 μg/mL.

## 4. Discussion

This study employed the ABTS, DPPH, and ORAC assays because they reflect distinct radical reaction mechanisms, ranging from electron donation to hydrogen atom transfer and peroxyl radical quenching, thereby providing a multidimensional antioxidant profile [35,36]. However, chemical scavenging tests alone cannot determine whether radical clearance translates into cellular protection. To address this limitation in the revised study, we combined chemical antioxidant screening with cell viability assessment, intracellular oxidative damage measurement, immunofluorescence analysis, and CQ-based western blot validation in neuronal-like SH-SY5Y cells and differentiated C2C12 myotubes, two cell models relevant to ageing-related oxidative and proteostatic stress [6,37]. Importantly, rather than focusing solely on EL, we included three additional plant extracts to establish a comparative framework. This screening strategy minimized selection bias and provided a rational basis for selecting EL as the most active candidate under identical experimental conditions [38], among plants that are abundant and traditionally used in Malaysia.

The antioxidant assays demonstrated that EL exhibited the strongest radical scavenging activity across all three systems, consistently yielding the lowest IC₅₀ values. This effect was most pronounced in the ORAC assay, where peroxyl radical quenching is particularly relevant to lipid peroxidation in biological membranes [35]. However, the DPPH regression slope did not reach statistical significance, indicating that the dose-response pattern in this assay should be interpreted cautiously. More importantly, the cellular data show that the biological interpretation should not rely on chemical assays alone. The CCK-8 results confirmed that EL at 100–300 μg/mL did not cause overt cytotoxicity after 24 h exposure, reducing the likelihood that the observed changes in oxidative damage and autophagy-related markers were secondary to nonspecific cell injury. In addition, the 8-OHdG ELISA results showed that EL reduced oxidative DNA damage in both SH-SY5Y and C2C12 cells, particularly at 200 and 300 μg/mL, while H_2_O_2_ markedly increased 8-OHdG levels. Since oxidative stress can damage nucleic acids and disrupt cellular homeostasis, these findings provide intracellular evidence supporting the redox-modulating effect of EL.

The immunofluorescence findings showed that EL increased LC3 puncta and reduced p62 fluorescence intensity in both cell models. LC3 puncta are commonly used as an indicator of autophagosome-associated structures, whereas p62 functions as an autophagy adaptor and substrate involved in selective cargo recognition [13]. These findings indicate that EL modulated autophagy-related marker profiles in neuronal-like and skeletal muscle cells. However, LC3 puncta and p62 fluorescence alone cannot distinguish between increased autophagosome formation and impaired downstream degradation. Therefore, these results should be interpreted as autophagy-related marker modulation rather than definitive evidence of complete autophagic flux activation.

To strengthen this interpretation, western blot analysis with chloroquine (CQ) was added as a lysosomal inhibition experiment. CQ alone increased LC3-II accumulation compared with the control group in both cell models, supporting effective lysosomal blockade. EL + CQ further increased LC3-II levels compared with CQ alone, particularly at 300 μg/mL in both cell lines and at 100 μg/mL in C2C12 cells. This pattern supports the interpretation that EL enhanced autophagy-related LC3-II turnover under lysosomal inhibition, rather than merely increasing a static LC3 signal under basal conditions [29]. At the same time, p62 results require cautious interpretation. Although EL reduced basal p62 abundance, CQ alone also reduced p62 levels, which differs from the canonical expectation that lysosomal inhibition often leads to p62 accumulation [39]. This non-canonical response may reflect cell-context-dependent regulation of p62 through transcriptional, proteasomal, stress-related, or selective autophagy-associated mechanisms [40,41]. Thus, p62 was considered a supportive autophagy-related marker rather than a definitive indicator of autophagic flux.

While this study supports antioxidant-related and autophagy-associated effects of EL, certain mechanistic limitations must be noted. Although CQ-based western blotting provided additional support for LC3-II turnover, complete autophagic flux was not assessed using tandem fluorescent LC3 reporters or direct lysosomal activity assays. In addition, 8-OHdG was used as an oxidative DNA damage endpoint, but further intracellular ROS or mitochondrial ROS measurements would provide a broader redox profile. The study also did not directly measure signaling pathways such as AMPK/mTOR, TFEB, or Keap1/Nrf2/p62, which have been implicated in redox-sensitive autophagy regulation [10,13,42]. Therefore, these pathways should be discussed only as possible mechanistic contexts rather than confirmed mechanisms. Future work should include pathway-specific validation, lysosomal functional assays, and in vivo studies to clarify whether EL directly regulates antioxidant-autophagy signaling.

## 5. Conclusions

In summary, this study shows a distinct hierarchy of in vitro antioxidant activity among the four plant extracts, with EL being the most potent, followed by LP, PM, and IA. Furthermore, EL did not induce overt cytotoxicity at the tested concentrations, reduced oxidative DNA damage, and modulated LC3 and p62 autophagy-related markers in both SH-SY5Y and C2C12 cells. Western blot analysis under CQ-mediated lysosomal inhibition further supported an effect of EL on LC3-II turnover, particularly at 300 μg/mL.The convergence of these properties suggests that EL may support cellular redox balance and autophagy-related proteostatic responses in neuronal-like and skeletal muscle cell models. Further studies are required to confirm complete autophagic flux, define the underlying signaling mechanisms, and evaluate whether these cellular effects can be reproduced in more complex in vivo models.

## Supporting information

S1 Table CCK-8 analysis of relative cell viability in SH-SY5Y and C2C12 cells. (DOCX)

S2 Table ABTS radical scavenging activity and TEAC values of Malaysian plant extracts. (DOCX)

S3 Table DPPH radical scavenging activity and TEAC values of Malaysian plant extracts. (DOCX)

S4 Table ORAC antioxidant capacity of Malaysian plant extracts. (DOCX)

S5 Table 8-OHdG ELISA analysis in SH-SY5Y and C2C12 cells. (DOCX)

S6 Table Western blot analysis of LC3-II and p62 in SH-SY5Y and C2C12 cells. (DOCX)

S1 File Anonymized outgoing material transfer agreement for standardized plant extracts. (PDF)

## Abbreviations

AAPH: 2,2’-azobis(2-methylpropionamidine) dihydrochloride
ABTS: 2,2′-azino-bis(3-ethylbenzothiazoline-6-sulfonic acid)
AMPK: AMP-activated protein kinase
BSA: Bovine Serum Albumin
CQ: chloroquine
DAPI: 4’,6-diamidino-2-phenylindole
DMEM: Dulbecco’s Modified Eagle Medium
DMSO: Dimethyl Sulfoxide
DPPH: 2,2-diphenyl-1-picrylhydrazyl
EL: *Eurycoma longifolia*
FBS: Fetal Bovine Serum
HRP: horseradish peroxidase
IA: *Ipomoea aquatica*
IC₅₀: Half-Maximal Inhibitory Concentration
Keap1: Kelch-like ECH-associated protein 1
LC3: Microtubule-Associated Protein 1 Light Chain 3
LP: *Labisia pumila*
mTOR: Mammalian Target of Rapamycin
Nrf2: Nuclear Factor Erythroid 2–Related Factor 2
ORAC: Oxygen Radical Absorbance Capacity
PBS: Phosphate-Buffered Saline
PM: *Persicaria minor*
PMSF: phenylmethylsulfonyl fluoride
PVDF: polyvinylidene difluoride
p62/SQSTM1: Sequestosome-1
RIPA: radioimmunoprecipitation assay
ROS: Reactive Oxygen Species
SD: Standard Deviation
TEAC: Trolox Equivalent Antioxidant Capacity
TFEB: Transcription Factor EB
ULK1: UNC-51 Like Autophagy Activating Kinase 1
