# Supplementary material for: A double-duty champion: Eurycoma longifolia counters oxidative stress and unlocks autophagy to safeguard neurons and muscle cells
Source: PLoS One. 2026 Aug 10;21(8):e0355828. doi: 10.1371/journal.pone.0355828 (PMC13456280; doi:10.1371/journal.pone.0355828)
Supplement: Supplementary file 1 [file pone.0355828.s007.pdf]

S1 File. Anonymized outgoing material transfer agreement for standardized plant extracts.

OUTGOING MATERIAL TRANSFER AGREEMENT ["MTA"]

IMPORTANT: PLEASE READ CAREFULLY. THIS IS A LEGALLY BINDING CONTRACT

PERSONAL IDENTIFIERS REDACTED FOR PUBLICATION

(names, identification number, physical address, and organization registration number)

SCOPE OF USE:

This MTA is only for the transfer of one (1) type of **Material**, identified as:

i) Physta (*Eurycoma longifolia*) in dried extract form, 10 gm
ii) Biokesum (*Persicaria minor)* in dried extract form, 10 gm
iii) *Labisia pumila* in dried extract form, 10 gm
iv) *Ipomoea aquatica* in dried extract form, 10 gm

from the manufacturing facility of **BIOTROPICS** and is valid for up to (3) shipments only. The amount shall be communicated and agreed between **BIOTROPICS** and **AY** for the second and third shipment. **BIOTROPICS** agrees to provide this **Material** and related information and/or data to **AY** for testing, evaluation and research purposes.

TERMS AND CONDITIONS

By signing this MTA, **AY** agrees to comply with the terms and conditions set forth in this MTA.

BIOTROPICS will provide **AY** such of the **Material** requested by **AY** as is, in **BIOTROPICS**'s sole judgment, as reasonable and appropriate. Such **Material** as is supplied to **AY** will be accompanied by a copy of this MTA where, on the last page (**Annex 1**), the identity of the **Material** being supplied must be listed (without which, this MTA is considered null and void).

COMPLIANCE WITH LAWS

AY agrees to use the **Material** in a safe manner and in compliance with Federal, State, and local statues, ordinances and regulations of Malaysia and Europe member states. This MTA constitutes the entire agreement among the parties relating to the **Material** and research information and may not be modified except by a document signed by all the parties.

If the above terms are acceptable, please have two copies of this document signed by the Authorized Official of **AY** and return them to:

PERSONAL CONTACT INFORMATION REDACTED (name, physical address, email, and phone)

SIGNATURE AND SPECIFIC DATE REDACTED

SIGNATURES, PERSONAL NAMES, AND SPECIFIC DATES REDACTED
