# Supplementary material for: A double-duty champion: Eurycoma longifolia counters oxidative stress and unlocks autophagy to safeguard neurons and muscle cells
Source: PLoS One. 2026 Aug 10;21(8):e0355828. doi: 10.1371/journal.pone.0355828 (PMC13456280; doi:10.1371/journal.pone.0355828)
Supplement: Supplementary file 2 [file pone.0355828.s001.docx]

**S1 Table. CCK-8 analysis of relative cell viability in SH-SY5Y and C2C12 cells.**

| **Cell line** | **Group** | **Relative cell viability (%)** **(mean ± SD) (n = 3)** | **Reference group** | **P value** | **Significance** |
| --- | --- | --- | --- | --- | --- |
| SH-SY5Y | Negative control | 100.00 ± 4.44 | — | — | — |
| SH-SY5Y | Positive cytotoxicity control | 54.43 ± 4.32 | Negative control | <0.001 | *** |
| SH-SY5Y | EL 100 μg/mL | 98.73 ± 3.00 | Negative control | 0.979 | n.s. |
| SH-SY5Y | EL 200 μg/mL | 96.74 ± 3.56 | Negative control | 0.666 | n.s. |
| SH-SY5Y | EL 300 μg/mL | 93.94 ± 2.83 | Negative control | 0.202 | n.s. |
| C2C12 | Negative control | 100.00 ± 5.96 | — | — | — |
| C2C12 | Positive cytotoxicity control | 54.43 ± 3.92 | Negative control | <0.001 | *** |
| C2C12 | EL 100 μg/mL | 98.54 ± 4.80 | Negative control | 0.986 | n.s. |
| C2C12 | EL 200 μg/mL | 95.93 ± 5.43 | Negative control | 0.687 | n.s. |
| C2C12 | EL 300 μg/mL | 93.81 ± 3.06 | Negative control | 0.366 | n.s. |

Note: Relative cell viability was assessed after 24 h treatment and expressed as a percentage of the negative control. Data are presented as mean ± SD (n = 3 biological replicates). P values are from one-way ANOVA followed by Dunnett's post hoc test versus the negative control group. *P < 0.05, **P < 0.01, ***P < 0.001; n.s., not significant.
