# Supplementary material for: A double-duty champion: Eurycoma longifolia counters oxidative stress and unlocks autophagy to safeguard neurons and muscle cells
Source: PLoS One. 2026 Aug 10;21(8):e0355828. doi: 10.1371/journal.pone.0355828 (PMC13456280; doi:10.1371/journal.pone.0355828)
Supplement: Supplementary file 3 [file pone.0355828.s002.docx]

**S2 Table. ABTS radical scavenging activity and TEAC values of Malaysian plant extracts.**

| **Extract (Plant)** | **Scavenging Activity** **(µmol Trolox/g, mean ± SD)** **100 / 200 / 300 μg/mL** | **R²** | **t** | **P value** | **P significance** | **IC₅₀ (μg/mL)** | **IC₅₀ significance** |
| --- | --- | --- | --- | --- | --- | --- | --- |
| *Persicaria minor* | 57.06 ± 11.31 77.27 ± 5.35 99.52 ± 7.86 | 0.975 | 36.05 | 0.018 | * | 494.46 | # |
| *Eurycoma longifolia* | 59.99 ± 4.07 85.07 ± 5.20 107.59 ± 7.98 | 0.996 | 32.205 | 0.020 | * | 167.22 | # |
| *Labisia pumila* | 36.94 ± 4.06 54.85 ± 10.03 87.91 ± 9.30 | 0.971 | 5.827 | 0.108 | n.s. | 451.62 | n.s. |
| *Ipomoea aquatica* | 34.37 ± 4.15 55.91 ± 4.02 84.01 ± 9.05 | 0.994 | 13.107 | 0.048 | * | 477.82 | # |

Note: Scavenging activity values are presented as μmol Trolox/g at 100, 200, and 300 μg/mL, respectively (mean ± SD, n = 3). R² denotes the coefficient of determination. The t value was calculated as slope/SE(slope) from linear regression. P value represents the statistical significance of the regression slope. *P < 0.05, **P < 0.01; n.s., not significant. IC₅₀ values are expressed as μg/mL, where lower IC₅₀ values indicate stronger antioxidant activity. #P < 0.05, ##P < 0.01 for IC₅₀ curve fitting; n.s., not significant.
