# Supplementary material for: A double-duty champion: Eurycoma longifolia counters oxidative stress and unlocks autophagy to safeguard neurons and muscle cells
Source: PLoS One. 2026 Aug 10;21(8):e0355828. doi: 10.1371/journal.pone.0355828 (PMC13456280; doi:10.1371/journal.pone.0355828)
Supplement: Supplementary file 4 [file pone.0355828.s003.docx]

**S3 Table. DPPH radical scavenging activity and TEAC values of Malaysian plant extracts.**

| **Extract (Plant)** | **Scavenging Activity** **(µmol Trolox/g, mean ± SD)** **100 / 200 / 300 μg/mL** | **R²** | **t** | **P value** | **P significance** | **IC₅₀ (μg/mL)** | **IC₅₀ significance** |
| --- | --- | --- | --- | --- | --- | --- | --- |
| *Persicaria minor* | 12.18 ± 1.34 17.24 ± 1.85 22.86 ± 0.61 | 0.9991 | 33.04 | 0.019 | * | 297.41 | # |
| *Eurycoma longifolia* | 13.07 ± 1.58 22.38 ± 1.52 27.28 ± 1.29 | 0.9688 | 5.57 | 0.113 | n.s. | 97.64 | n.s. |
| *Labisia pumila* | 8.42 ± 1.68 14.19 ± 1.28 21.68 ± 1.86 | 0.9944 | 13.33 | 0.048 | * | 312.14 | # |
| *Ipomoea aquatica* | 9.93 ± 1.38 14.73 ± 1.47 22.46 ± 1.16 | 0.9819 | 7.37 | 0.086 | n.s. | 313.06 | n.s. |

Note: Scavenging activity values are presented as μmol Trolox/g at 100, 200, and 300 μg/mL, respectively (mean ± SD, n = 3). R² denotes the coefficient of determination. The t value was calculated as slope/SE(slope) from linear regression. P value represents the statistical significance of the regression slope. *P < 0.05, **P < 0.01; n.s., not significant. IC₅₀ values are expressed as μg/mL, where lower IC₅₀ values indicate stronger antioxidant activity. #P < 0.05, ##P < 0.01 for IC₅₀ curve fitting; n.s., not significant.
