# Supplementary material for: A double-duty champion: Eurycoma longifolia counters oxidative stress and unlocks autophagy to safeguard neurons and muscle cells
Source: PLoS One. 2026 Aug 10;21(8):e0355828. doi: 10.1371/journal.pone.0355828 (PMC13456280; doi:10.1371/journal.pone.0355828)
Supplement: Supplementary file 5 [file pone.0355828.s004.docx]

**S4 Table. ORAC antioxidant capacity of Malaysian plant extracts.**

| **Extract (Plant)** | **Scavenging Activity** **(µmol Trolox/g, mean ± SD)** **100 / 200 / 300 μg/mL** | **R²** | **t** | **P value** | **P significance** | **IC₅₀ (μg/mL)** | **IC₅₀ significance** |
| --- | --- | --- | --- | --- | --- | --- | --- |
| *Persicaria minor* | 31.79 ± 4.20 55.28 ± 6.13 70.38 ± 2.09 | 0.984 | 7.967 | 0.079 | n.s. | 339.42 | n.s. |
| *Eurycoma longifolia* | 38.21 ± 5.80 59.81 ± 5.90 81.12 ± 4.81 | 0.988 | 256.284 | 0.002 | ** | 67.90 | ## |
| *Labisia pumila* | 25.47 ± 5.42 45.19 ± 6.63 62.55 ± 3.98 | 0.997 | 27.214 | 0.023 | * | 83.72 | # |
| *Ipomoea aquatica* | 26.42 ± 3.50 42.08 ± 4.27 65.47 ± 4.50 | 0.987 | 8.745 | 0.072 | n.s. | 480.96 | n.s. |

Note: Scavenging activity values are presented as μmol Trolox/g at 100, 200, and 300 μg/mL, respectively (mean ± SD, n = 3). R² denotes the coefficient of determination. The t value was calculated as slope/SE(slope) from linear regression. P value represents the statistical significance of the regression slope. *P < 0.05, **P < 0.01; n.s., not significant. IC₅₀ values are expressed as μg/mL, where lower IC₅₀ values indicate stronger antioxidant activity. #P < 0.05, ##P < 0.01 for IC₅₀ curve fitting; n.s., not significant.
