# Supplementary material for: A double-duty champion: Eurycoma longifolia counters oxidative stress and unlocks autophagy to safeguard neurons and muscle cells
Source: PLoS One. 2026 Aug 10;21(8):e0355828. doi: 10.1371/journal.pone.0355828 (PMC13456280; doi:10.1371/journal.pone.0355828)
Supplement: Supplementary file 6 [file pone.0355828.s005.docx]

**S5 Table. 8-OHdG ELISA analysis in SH-SY5Y and C2C12 cells.**

| **Cell line** | **Group** | **8-OHdG (ng/mL)** **(mean ± SD) (n = 3)** | **Reference group** | **P value** | **Significance** |
| --- | --- | --- | --- | --- | --- |
| SH-SY5Y | Control | 2.98 ± 0.15 | — | — | — |
| SH-SY5Y | EL 100 μg/mL | 2.60 ± 0.11 | Control | 0.055 | n.s. |
| SH-SY5Y | EL 200 μg/mL | 2.45 ± 0.12 | Control | 0.009 | ** |
| SH-SY5Y | EL 300 μg/mL | 2.33 ± 0.17 | Control | 0.002 | ** |
| SH-SY5Y | H₂O₂ 100 μM | 6.11 ± 0.24 | Control | <0.001 | *** |
| C2C12 | Control | 2.91 ± 0.14 | — | — | — |
| C2C12 | EL 100 μg/mL | 2.57 ± 0.13 | Control | 0.062 | n.s. |
| C2C12 | EL 200 μg/mL | 2.45 ± 0.11 | Control | 0.014 | * |
| C2C12 | EL 300 μg/mL | 2.33 ± 0.15 | Control | 0.003 | ** |
| C2C12 | H₂O₂ 100 μM | 5.86 ± 0.21 | Control | <0.001 | *** |

Note: 8-OHdG levels were measured by ELISA after treatment with EL or H₂O₂. Data are presented as mean ± SD (n = 3 biological replicates). P values are from one-way ANOVA followed by Dunnett's post hoc test versus the control group. *P < 0.05, **P < 0.01, ***P < 0.001; n.s., not significant.
