# Supplementary material for: A double-duty champion: Eurycoma longifolia counters oxidative stress and unlocks autophagy to safeguard neurons and muscle cells
Source: PLoS One. 2026 Aug 10;21(8):e0355828. doi: 10.1371/journal.pone.0355828 (PMC13456280; doi:10.1371/journal.pone.0355828)
Supplement: Supplementary file 7 [file pone.0355828.s006.docx]

**S6 Table. Western blot analysis of LC3-II and p62 in SH-SY5Y and C2C12 cells.**

| **Cell line** | **Protein** | **Group** | **Relative protein level** **(mean ± SD) (n = 3)** | **Reference group** | **P value** | **Significance** |
| --- | --- | --- | --- | --- | --- | --- |
| SH-SY5Y | LC3-II | Ctrl | 1.000 ± 0.057 | — | — | — |
| SH-SY5Y | LC3-II | EL 100 μg/mL | 1.666 ± 0.152 | Ctrl | 0.010 | * |
| SH-SY5Y | LC3-II | EL 300 μg/mL | 2.023 ± 0.254 | Ctrl | 0.012 | * |
| SH-SY5Y | LC3-II | CQ 50 μM | 2.658 ± 0.144 | Ctrl | <0.001 | *** |
| SH-SY5Y | LC3-II | EL 100 μg/mL + CQ | 3.330 ± 0.350 | CQ 50 μM | 0.172 | n.s. |
| SH-SY5Y | LC3-II | EL 300 μg/mL + CQ | 3.702 ± 0.300 | CQ 50 μM | 0.028 | * |
| SH-SY5Y | p62 | Ctrl | 1.000 ± 0.057 | — | — | — |
| SH-SY5Y | p62 | EL 100 μg/mL | 0.811 ± 0.053 | Ctrl | 0.066 | n.s. |
| SH-SY5Y | p62 | EL 300 μg/mL | 0.559 ± 0.080 | Ctrl | 0.007 | ** |
| SH-SY5Y | p62 | CQ 50 μM | 0.331 ± 0.034 | Ctrl | <0.001 | *** |
| SH-SY5Y | p62 | EL 100 μg/mL + CQ | 0.519 ± 0.047 | CQ 50 μM | 0.024 | * |
| SH-SY5Y | p62 | EL 300 μg/mL + CQ | 0.687 ± 0.071 | CQ 50 μM | 0.007 | ** |
| C2C12 | LC3-II | Ctrl | 1.000 ± 0.125 | — | — | — |
| C2C12 | LC3-II | EL 100 μg/mL | 1.736 ± 0.129 | Ctrl | 0.010 | * |
| C2C12 | LC3-II | EL 300 μg/mL | 2.190 ± 0.207 | Ctrl | 0.005 | ** |
| C2C12 | LC3-II | CQ 50 μM | 2.583 ± 0.213 | Ctrl | 0.002 | ** |
| C2C12 | LC3-II | EL 100 μg/mL + CQ | 3.501 ± 0.238 | CQ 50 μM | 0.037 | * |
| C2C12 | LC3-II | EL 300 μg/mL + CQ | 4.045 ± 0.249 | CQ 50 μM | 0.008 | ** |
| C2C12 | p62 | Ctrl | 1.000 ± 0.086 | — | — | — |
| C2C12 | p62 | EL 100 μg/mL | 0.817 ± 0.056 | Ctrl | 0.168 | n.s. |
| C2C12 | p62 | EL 300 μg/mL | 0.646 ± 0.077 | Ctrl | 0.029 | * |
| C2C12 | p62 | CQ 50 μM | 0.397 ± 0.036 | Ctrl | 0.002 | ** |
| C2C12 | p62 | EL 100 μg/mL + CQ | 0.608 ± 0.076 | CQ 50 μM | 0.058 | n.s. |
| C2C12 | p62 | EL 300 μg/mL + CQ | 0.709 ± 0.047 | CQ 50 μM | 0.004 | ** |

Note: Protein levels were normalized to β-actin and expressed relative to the control group. Data are presented as mean ± SD (n = 3 biological replicates). P values are from one-way ANOVA followed by Šídák-corrected planned comparisons. Under basal conditions, EL-treated groups and the CQ-only group were compared with the control group; under lysosomal inhibition, EL + CQ groups were compared with the CQ-only group. *P < 0.05, **P < 0.01, ***P < 0.001; n.s., not significant.
